# Persistent Misperceptions about Nicotine among US Physicians: Results from a Randomized Survey Experiment

**DOI:** 10.3390/ijerph18147713

**Published:** 2021-07-21

**Authors:** Michelle T. Bover Manderski, Michael B. Steinberg, Olivia A. Wackowski, Binu Singh, William J. Young, Cristine D. Delnevo

**Affiliations:** 1 Department of Biostatistics & Epidemiology, Rutgers School of Public Health, Piscataway, NJ 08854, USA; 2 Rutgers Center for Tobacco Studies, New Brunswick, NJ 08901, USA; wackowol@cts.rutgers.edu (O.A.W.); bs649@cts.rutgers.edu (B.S.); william.j.young@rutgers.edu (W.J.Y.); delnevo@cts.rutgers.edu (C.D.D.); 3 Department of Medicine, Rutgers Robert Wood Johnson Medical School, New Brunswick, NJ 08901, USA; steinbmb@rwjms.rutgers.edu; 4 Rutgers Tobacco Dependence Program, New Brunswick, NJ 08901, USA; 5 Department of Health Behavior, Society & Policy, Rutgers School of Public Health, Piscataway, NJ 08854, USA

**Keywords:** nicotine, perceptions, survey, physicians

## Abstract

We conducted a survey experiment among US physicians to evaluate whether question wording impacted perceptions about the health effects of nicotine. 926 physicians were randomized to receive one of two versions of a question matrix that asked about the “extent to which they agree or disagree that ‘nicotine’ (Version 1) or ‘nicotine, on its own,’ (Version 2) directly contributes to” birth defects, cardiovascular disease (CVD), cancer, depression, and chronic obstructive pulmonary disease (COPD). We evaluated whether question condition predicted strong agreement and/or agreement with each statement, and assessed demographic correlates of each outcome while adjusting for question version. Physicians who received Version 2 were less likely to “strongly agree” that nicotine directly caused birth defects (Prevalence Ratio (PR) 0.84, 95% CI 0.72–0.98), CVD (PR 0.89, 95% CI 0.84–0.95), cancer (PR 0.81, 95% CI 0.75–0.87), and COPD (PR 0.78, 95% CI 0.72–0.84). Females were more likely to “strongly agree” that nicotine directly contributes to birth defects and cancer, and family physicians were most likely to “strongly agree” that nicotine directly contributes to CVD, cancer, and COPD. Question wording is important when measuring physicians’ beliefs about nicotine; however, even after accounting for question version, misperceptions about the direct health effects of nicotine were common and varied by sex and specialty.

## 1. Introduction

The health consequences of tobacco use are extensive and known to include numerous cancers, cardiovascular disease (CVD), chronic obstructive pulmonary disease (COPD), and pregnancy complications [1]. Most tobacco-related disease is attributed to the numerous toxicants present in tobacco and tobacco smoke [1,2]. However, as the addictive component of tobacco products, the effects of nicotine are often conflated with the effects of other tobacco constituents. The situation is complicated by the wide-range of nicotine containing products, and varying levels of nicotine delivered among them. Indeed, many in the general population misperceive nicotine as responsible for smoking-related health risks, including cancer [3,4,5,6]. For example, a survey reporting on data from two waves (2015 and 2017) of a large US national survey found that 51% of participants agreed that “nicotine is the substance that causes most of the cancer caused by smoking” [7]. Another 2016 survey with adults ages 18–40 found that 66% believed that a relatively large or very large part of the “health risks of smoking” come from nicotine, and 60% believed that nicotine was responsible for a relatively large/very large part “of the cancer caused by smoking” [6].

One might expect this misperception to be limited to the general population, but research studies have also documented such misperception among healthcare professionals. For example, a 2007 study found that 60% of nurses incorrectly perceived nicotine as carcinogenic [8]. Indeed, our own national survey of US physicians in 2018 found that a majority of doctors ‘strongly agreed’ that “nicotine directly contributes” to the development of CVD, COPD, and cancer [9]. Additionally, we found that misperceptions regarding nicotine’s role differed by physician specialty, with pulmonologists generally being least likely to hold nicotine misperceptions while family physicians were more likely than most other specialists to hold nicotine misperceptions. Similar findings of nicotine misperceptions have been noted among health care providers in other countries [10].

A growing body of survey methods research highlights the importance of question wording and format when assessing perceptions [11,12,13]. As such, we considered that the wording of survey questions about nicotine health effects may have contributed to our finding of prevalent nicotine misperceptions. For example, it is possible that some respondents may have answered by thinking about the health effects of tobacco use that are mediated by nicotine addiction rather than nicotine itself (i.e., nicotine causes addiction to smoking, which then leads to lung cancer). Moreover, survey satisficing—wherein respondents put forth the minimum amount of effort necessary to provide an acceptable response—has been posited as an explanation for acquiescence bias, or the tendency to agree with assertions contained in a question [14]. If some of our respondents were satisficing, they may not have read the question with enough care in order to distinguish between nicotine and cigarettes more generally, contributing to the high amount of agreement we uncovered.

For these reasons, the wording of questions about nicotine’s health effects may impact how physicians report their perceptions on this topic. To evaluate this hypothesis, we conducted a randomized survey experiment to explore the effect of question wording on estimates of nicotine misperception among a national sample of US physicians.

## 2. Materials and Methods

We embedded a randomized split-sample experiment about nicotine risk perception questions in a 2019 national survey of US physicians designed to explore physicians’ knowledge and communication with patients about tobacco and electronic nicotine vapor products. Random samples of 750 physicians in each of three specialties (family medicine, internal medicine, and obstetrics/gynecology (OBGYN)) were selected from the AMA physician master file. To be eligible, physicians must have been board certified in their specialty and actively seeing outpatients. Sampled physicians were mailed a letter that included a link to the web-based survey, as well as two additional reminder mailings. In a fourth and final mailing, sampled physicians who had not yet responded were provided with a paper copy of the survey to return by postal mail. In total, 995 physicians participated in the study and 305 were ineligible, yielding an AAPOR response rate of 57.8%. The present analysis is restricted to 926 respondents who completed by web (and thus able to be randomized to the online experiment).

The survey included domains about demographic characteristics, treatment practices, and knowledge/perceptions about tobacco and electronic nicotine products (e.g., e-cigarettes). Participants were randomly assigned to receive one of two versions of a question matrix about health effects of nicotine: Version 1 (from our previous survey [9], “Please indicate the extent to which you agree or disagree that nicotine directly contributes to the development of the following health problems by selecting your choice” and Version 2, “Please indicate the extent to which you agree or disagree that nicotine on its own directly contributes to the development of the following health problems by selecting your choice.” Both versions then presented response options “Strongly Agree,” “Agree,” “Disagree,” and “Strongly Disagree” corresponding to each of five health outcomes: birth defects, cardiovascular disease, cancer, depression, and COPD (See online supplement, Figure S1).

SAS software version 9.4 (SAS Institute, Cary, NC) were used for all analyses. We used descriptive statistics to characterize the study sample and compared demographics across survey condition to assess the success of randomization. To compare the distribution of responses by question version, we assigned numeric values ranging from 1 (Strongly Disagree) to 4 (Strongly Agree) and calculated the mean response for each health effect. Since the distributions were highly skewed, we compared response means using two-sided Wilcoxon Rank-Sum tests. We then estimated prevalence ratios adjusted for gender, age, and specialty using log-binomial regression to describe the association between question version and “strongly agreeing” that nicotine directly contributes to each health effect. We separately assessed effect modification by physician specialty and age by including a cross-product term (e.g., Version*Specialty, Version*Age) in the regression models. We repeated the regression analyses modeling the prevalence of “agreeing or strongly agreeing” as a function of question version, age, gender, and specialty.

## 3. Results

Of the 926 web-survey participants, 461 and 465 were randomized to Version 1 and Version 2, respectively. Version assignment did not differ by age, gender, race/ethnicity, graduation year, or specialty (Table 1). Overall, about half (50.6%) of respondents were female, two-thirds (66.8%) were non-Hispanic white, and the median age was 51 years.

For the outcomes of birth defects, cardiovascular disease, cancer, and COPD, the distribution of responses to the nicotine health effects questions differed significantly by survey version, such that the response means were lower (towards strong disagreement) among the group that received Version 2 (Table 2). In addition, smaller proportions of respondents in the Version 2 group relative to the Version 1 group “strongly agreed” that nicotine directly contributed to each health effect, with marked differences for the questions about cancer (69.6% vs. 85.0%) and COPD (67.3% vs. 85.2%).

**Figure 1:**
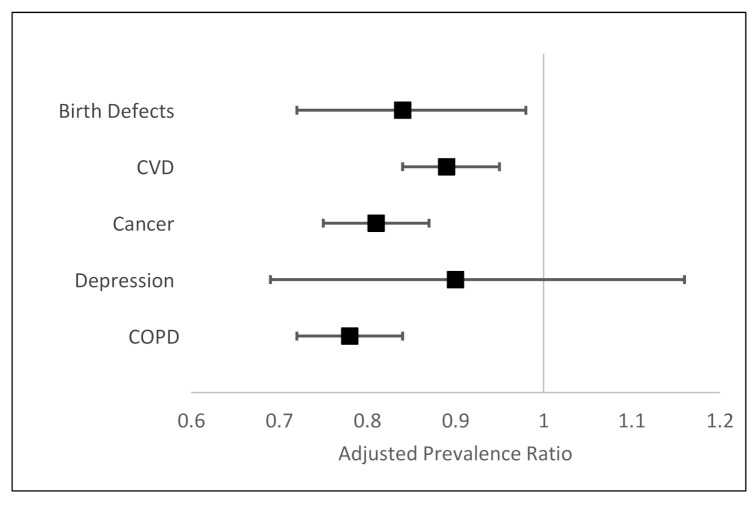
Association between question version and “strongly agreeing” that nicotine directly contributes to each health effect. Adjusted for age, gender, and specialty.

In regression analyses adjusted for gender, age, and specialty, physicians who received Version 2 were significantly less likely to “strongly agree” that nicotine directly caused birth defects (PR 0.84, 95% CI 0.72–0.98), CVD (PR 0.89, 95% CI 0.84–0.95), cancer (PR 0.81, 95% CI 0.75–0.87), and COPD (PR 0.78, 95% CI 0.72–0.84) (Table 3, Figure 1). We did not find evidence of effect modification by specialty or age (all *p*-values for interaction >0.1, data not shown). The second set of regression models, modeling prevalence of “strongly agree or agree” versus “disagree or strongly disagree,” also found that physicians who received Version 2 were significantly less likely to perceive that nicotine directly contributes to CVD, cancer, and COPD, although these associations were not as strong (Table 4). The association between question version and agreeing that nicotine directly contributes to birth defects was attenuated and not significant (PR 0.94, 95% CI 0.88–1.01).

Female physicians were significantly more likely to “strongly agree” that nicotine directly contributes to birth defects (PR 1.19, 95% CI 1.01–1.39) and cancer (PR 1.08 95% CI 1.01–1.15), even after adjusting for questionnaire version, age, and medical specialty (Table 3). However, when modeling “strongly agree or agree” as the outcome, sex was not significantly associated with perceptions about nicotine’s direct contribution to any outcome.

Differences in perceptions were noted across specialty groups. For example, family medicine and internal medicine physicians were 74% (PR 1.74, 95% CI 1.41–2.13) and 69% (PR 1.69, 95% CI 1.35–2.11) more likely than OB/GYN specialists to “strongly agree” that nicotine directly contributes to birth defects, respectively (Table 3). Family physicians were most likely to “strongly agree” that nicotine directly contributes to CVD, cancer, and COPD. In the second set of regression models, the associations between specialty group and “strongly agreeing or agreeing” that nicotine causes birth defects, CVD, cancer, and COPD were attenuated but still significant (Table 4).

## 4. Discussion

In a follow-up to our prior research that observed misperceptions about the health effects of nicotine among a national sample of US physicians [9], we sought to assess the impact of question wording on estimates of physician perceptions about nicotine using a randomized split-sample survey experiment. Indeed, question version was significantly associated with “strongly agreeing” that nicotine directly contributes to development of birth defects, CVD, cancer, and COPD, even after adjusting for age, sex, and medical specialty. Specifically, we found that physicians who answered a question version that more explicitly asked about the effects of nicotine “on its own” reported lower levels of misperceptions about the health effects caused by nicotine, suggesting the potential importance of this type of wording. Question version was still relevant when modeling “strongly agree or agree” as the outcome, suggesting that question wording is important for distinguishing agreement versus disagreement generally, as well as strong agreement versus moderate agreement or disagreement.

Even after accounting for question version, the proportion of surveyed physicians who believe that nicotine directly contributes to these health outcomes is alarmingly high. It is possible that participants are conflating the addictive effect of nicotine with the comparatively more harmful effects of tobacco use. This overestimation of the harms caused by nicotine may be exacerbated by a potential availability bias, as previous research has demonstrated that nicotine is the cigarette constituent most familiar to many people [15]. As such, respondents may have made judgements and overestimations about the probability that nicotine causes harm based on the ease with which they associate nicotine and cigarettes more generally [16]. We also observed differences in nicotine perceptions by gender and specialty. In general, females were more likely to endorse “strong agreement” that nicotine directly contributes to each health effect; however, these associations did not persist when modeling the more general “agree vs. disagree” outcome. Family medicine physicians were most likely to misperceive nicotine as a direct contributor to CVD, cancer, and COPD regardless of how the outcome was defined.

Our findings are consistent with our previous study. For example, our previous analysis of data collected during 2018 also found that females were more likely than males to misperceive nicotine as a direct contributor to CVD, cancer, and COPD; however, the magnitude of these associations were somewhat weaker in the present study than it was in 2018 (e.g., PR, females vs. males, for strongly agreeing that nicotine directly contributes to CVD was 1.10 (95% CI 1.04–1.17) previously and 1.04 (95% CI 0.98–1.10) currently). Future surveillance of physician perceptions across demographics and specialties is warranted to understand whether this tightening “gender gap” will continue.

These findings should be interpreted in the light of some limitations that are common to survey research. First, our findings that perceptions about nicotine vary by physician gender and specialty may be subject to selection bias if physicians who chose to participate in the survey differ from those who did not with respect to their perceptions about nicotine. However, sample representativeness is unlikely to threaten the validity of the finding that physician perceptions are impacted by question version, given that question version was randomly assigned and there were no differences in demographics or medical specialty across study groups. Second, participants received only one of the two different question versions when they completed the survey; inclusion of additional question versions in the experiment may have been more helpful for learning about how to best measure nicotine perceptions. Future planned studies will include version of the question explicitly emphasizing potential non-addiction-related effects. Additionally, future qualitative research may be useful for elucidating the underlying beliefs and reasons for the observed response patterns among physicians. Finally, although the survey sample was selected at random within specialty groups, results are not weighted to be representative of all family medicine, internal medicine, and OB/GYN physicians in the U.S.

Despite these limitations, this study of US physicians from several specialties demonstrates that question wording is important when assessing physician beliefs about nicotine health effects, and this finding would be likely to extend to surveys about physician perspectives about other topics as well. In addition, we found that even after accounting for question version, perceiving that nicotine directly contributes to certain health effects was very common, and there were differences in perceptions by sex and medical specialty. This highlights the importance of improving physician education about tobacco and nicotine, which may improve patient-provider communications about tobacco use, tobacco cessation, and use of nicotine containing products.

## 5. Conclusions

Our findings demonstrate that question wording is important when measuring physician beliefs about nicotine. However, even after accounting for question version, misperceptions about the direct health effects of nicotine were common, and perceptions about nicotine varied by sex and specialty. Provider education about tobacco and nicotine should be prioritized in order to optimize patient–provider communications about tobacco use and cessation.

## Figures and Tables

**Table 1:** Sample Characteristics by Question Version, (N = 926).

	Overall (N = 926) ^a^		Question Version				
			Version 1 (n = 461)		Version 2 (n = 465)		*p*-Value ^b^
	*n*	%	*n*	%	*n*	%	
Age, years ^c^							
Median (IQR)	51.0	(15.0)	51.0	(15.0)	52.0	(16.0)	0.6586
Gender							
Female	450	50.6%	221	49.4%	229	51.7%	0.5016
Male	440	49.4%	226	50.6%	214	48.3%	
Race/Ethnicity							
Non-Hispanic White	592	66.8%	289	64.7%	303	69.0%	0.1356
Non-Hispanic Black	44	5.0%	30	6.7%	14	3.2%	
Hispanic	41	4.6%	18	4.0%	23	5.2%	
Non-Hispanic Asian/PI	108	12.2%	53	11.9%	55	12.5%	
Non-Hispanic South Asian	55	6.2%	31	6.9%	24	5.5%	
Non-Hispanic Other	46	5.2%	26	5.8%	20	4.6%	
Graduation year ^d^							
Median (IQR)	1995.0	(17.0)	1996.0	(16.0)	1994.0	(17.0)	0.6097
Specialty							
Family Medicine	343	37.0%	178	38.6%	165	35.5%	0.5746
Internal Medicine	255	27.5%	126	27.3%	129	27.7%	
OB/GYN	328	35.4%	157	34.1%	171	36.8%	

SD, Standard Deviation; IQR, Interquartile Range. ^a^ Frequencies may not total 926 due to item nonresponse. ^b^ Characteristics compared by question version using Pearson chi-square (for categorical variables) or two-sided Wilcoxon Rank-Sum (for continuous variables) test. ^c^ Imputed for 7 respondents as median age within same specialty and graduation year. ^d^ Imputed for 21 respondents as median year within same specialty and age.

**Table 2:** Distribution of Responses to Nicotine Questions, by Question Version (N = 926).

	Question Version				
	Version 1 (n = 461)		Version 2 (n = 465)		*p*-Value ^b^
	*n*	%	*n*	%	
Birth Defects					
Mean ^a^ ± SD	3.14	±0.97	3.01	±0.98	0.0247
Strongly Disagree	37	8.1%	42	9.1%	
Disagree	76	16.6%	92	19.9%	
Agree	131	28.6%	148	32.0%	
Strongly Agree	214	46.7%	180	39.0%	
CVD					
Mean ^a^ ± SD	3.78	±0.64	3.64	±0.79	0.0005
Strongly Disagree	13	2.8%	22	4.7%	
Disagree	15	3.3%	24	5.2%	
Agree	32	6.9%	55	11.9%	
Strongly Agree	401	87.0%	363	78.2%	
Cancer					
Mean ^a^ ± SD	3.70	±0.77	3.45	±0.93	<0.0001
Strongly Disagree	23	5.0%	30	6.5%	
Disagree	21	4.6%	52	11.2%	
Agree	25	5.4%	59	12.7%	
Strongly Agree	392	85.0%	322	69.6%	
Depression					
Mean ^a^ ± SD	2.82	±0.87	2.79	±0.85	0.5378
Strongly Disagree	32	7.1%	32	7.0%	
Disagree	121	26.7%	127	27.7%	
Agree	196	43.3%	206	44.9%	
Strongly Agree	104	23.0%	94	20.5%	
COPD					
Mean ^a^ ± SD	3.69	±0.80	3.36	±1.02	<0.0001
Strongly Disagree	23	5.0%	42	9.1%	
Disagree	27	5.9%	62	13.4%	
Agree	18	3.9%	47	10.2%	
Strongly Agree	391	85.2%	311	67.3%	

^a^ Strongly agree = 4, Agree = 3, Disagree = 2, Strongly Disagree = 1. ^b^ 2-sided Wilcoxon Rank-Sum Test.

**Table 3:** Prevalence of and multivariable associations with “strongly agreeing” that nicotine directly contributes to each health effect.

	Birth Defects				CVD				Cancer				Depression				COPD			
	%	aPR	(95% CI)		%	aPR	(95% CI)		%	aPR	(95% CI)		%	aPR	(95% CI)		%	aPR	(95% CI)	
Question Version																				
Original	46.7%	Ref.			87.0%	Ref.			85.0%	Ref.			23.0%	Ref.			85.2%	Ref.		
Modified	39.0%	0.84	(0.72, 0.98)		78.2%	0.89	(0.84, 0.95)		69.6%	0.81	(0.75, 0.87)		20.5%	0.90	(0.69, 1.16)		67.3%	0.78	(0.72, 0.84)	
Sex																				
Male	39.9%	Ref.			79.5%	Ref.			73.9%	Ref.			21.8%	Ref.			72.5%	Ref.		
Female	43.9%	1.19	(1.01, 1.39)		84.8%	1.04	(0.98, 1.10)		79.8%	1.08	(1.01, 1.15)		20.4%	0.91	(0.69, 1.19)		78.7%	1.06	(0.99, 1.14)	
Age																				
Per 5 Years	---	0.97	(0.94, 1.01)		---	1.00	(0.99, 1.02)		---	1.00	(0.99, 1.02)		---	0.96	(0.90, 1.03)		---	1.01	(0.99, 1.02)	
Specialty																				
Family Medicine	51.0%	1.74	(1.41, 2.13)		89.2%	Ref.			82.2%	Ref.			22.2%	Ref.			81.1%	Ref.		
Internal Medicine	48.4%	1.69	(1.35, 2.11)		75.6%	0.84	(0.78, 0.92)		71.2%	0.86	(0.78, 0.94)		22.8%	1.05	(0.76, 1.46)		68.9%	0.85	(0.77, 0.94)	
OB/GYN	29.9%	Ref.			81.1%	0.92	(0.87, 0.98)		76.8%	0.94	(0.88, 1.01)		20.4%	0.94	(0.69, 1.29)		76.9%	0.95	(0.89, 1.02)	

aPR, Prevalence Ratio (adjusted for all variables in table); CI, Confidence Interval.

**Table 4:** Prevalence of and multivariable associations with “agreeing” or “strongly agreeing” that nicotine directly contributes to each health effect.

	Birth Defects				CVD				Cancer				Depression				COPD			
	%	aPR	(95% CI)		%	aPR	(95% CI)		%	aPR	(95% CI)		%	aPR	(95% CI)		%	aPR	(95% CI)	
Question Version																				
Original	75.3%	Ref.			93.9%	Ref.			90.5%	Ref.			66.2%	Ref.			89.1%	Ref.		
Modified	71.0%	0.94	(0.88, 1.01)		90.1%	0.95	(0.92, 0.99)		82.3%	0.89	(0.85, 0.94)		65.4%	1.00	(0.91, 1.10)		77.5%	0.86	(0.81, 0.91)	
Sex																				
Male	71.1%	Ref.			88.9%	Ref.			82.8%	Ref.			61.6%	Ref.			79.7%	Ref.		
Female	74.0%	1.07	(1.00, 1.16)		94.6%	1.04	(1.00, 1.08)		88.9%	1.04	(0.99, 1.09)		68.7%	1.08	(0.98, 1.19)		85.8%	1.05	(0.99, 1.11)	
Age																				
Per 5 Years	---	0.99	(0.97, 1.01)		---	1.00	(0.99, 1.01)		---	1.00	(0.98, 1.01)		---	0.96	(0.93, 0.98)		---	1.00	(0.99, 1.02)	
Specialty																				
Family Medicine	84.0%	1.48	(1.33, 1.65)		94.5%	Ref.			90.1%	Ref.			72.2%	Ref.			86.6%	Ref.		
Internal Medicine	78.2%	1.40	(1.24, 1.59)		85.4%	0.92	(0.86, 0.98)		79.5%	0.88	(0.81, 0.95)		61.2%	0.88	(0.78, 1.00)		76.8%	0.89	(0.82, 0.97)	
OB/GYN	57.9%	Ref.			94.5%	1.01	(0.98, 1.04)		87.8%	0.97	(0.93, 1.02)		62.7%	0.87	(0.78, 0.97)		84.9%	0.99	(0.93, 1.04)	

aPR, Prevalence Ratio (adjusted for all variables in table); CI, Confidence Interval.

## Data Availability

The data presented in this study are available on reasonable request from the corresponding author.

## Supplementary Materials

The following are available online at https://www.mdpi.com/article/10.3390/ijerph18147713/s1, Figure S1: Exact wording and format of experimental question, versions 1 and 2.
