# Supplementary figures and images for: Persistent Misperceptions about Nicotine among US Physicians: Results from a Randomized Survey Experiment
Source: Int J Environ Res Public Health. 2021 Jul 21;18(14):7713. doi: 10.3390/ijerph18147713 (PMC8306881; doi:10.3390/ijerph18147713)

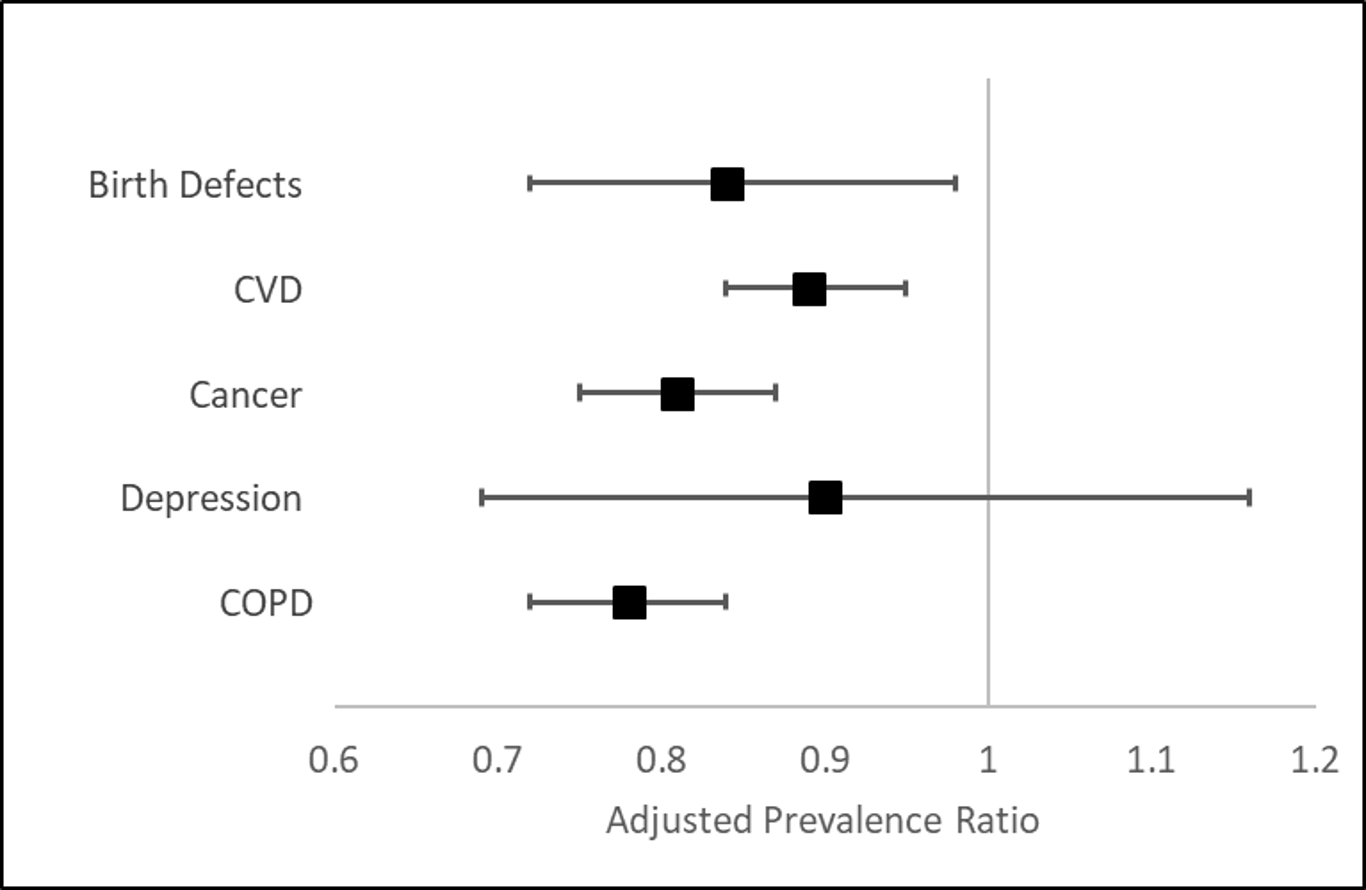

Supplement: Supplementary file 1 [file ijerph-18-07713-s001.zip › ijerph-1288526-supplementary.png]
